# Evolution of quasi-bound states in the circular n–p junction of bilayer graphene under magnetic field

**DOI:** 10.1038/s41598-020-73377-6

**Published:** 2020-10-01

**Authors:** Haijiao Ji, Yueting Pan, Haiwen Liu

**Affiliations:** Department of Physics, Beijing Normal University, Beijing, 100875 China

**Keywords:** Graphene, Electronic properties and devices, Mechanical and structural properties and devices

## Abstract

Electron in gapless bilayer graphene can form quasi-bound states when a circular symmetric potential is created in bilayer graphene. These quasi-bound states can be adjusted by tuning the radius and strength of the potential barrier. We investigate the evolution of quasi-bound states spectra in the circular n–p junction of bilayer graphene under the magnetic field numerically. The energy levels of opposite angular momentum split and the splitting increases with the magnetic field. Moreover, weak magnetic fields can slightly shift the energy levels of quasi-bound states. While strong magnetic fields induce additional resonances in the local density states, which originates from Landau levels. We demonstrate that these numerical results are consistent with the semiclassical analysis based on Wentzel–Kramers–Brillouin approximation. Our results can be verified experimentally via scanning tunneling microscopy measurements.

## Introduction

The successful fabrication of graphene has stimulated much interest in two-dimensional physics^1,2^. Graphene has many interesting phenomena, such as half-integer quantum Hall effects, resonant Klein tunneling, and Hofstadter butterflies^3–5^. However, Klein tunneling makes it becomes a challenge to create bound states in gapless graphene^6–9^. Recently, several experimental works have demonstrated that electrons can be trapped in nanometre-scale circular n–p junctions (CNPJs) on monolayer graphene, and the energy levels of the quasi-bound states (QBSs) in experiment closely matched the solution of massless Dirac equation^10–15^.

Researches on monolayer and multilayer graphene are progressing rapidly due to their interesting band structures, Berry phases, and characteristics of quasi-particles^16–22^. AB-stacked bilayer graphene (BLG), as a stable material, has attracted much attention. It is a gapless semiconductor with a chiral parabolic low-energy band structure^23,24^. Thus, the low-energy electrons in AB-stacked BLG does not satisfy the standard Dirac equation, suggesting that its QBSs have different energy spectra from those in monolayer graphene^22,25–31^. Many previous studies have focused on the real bound states in gapped BLG theoretically and experimentally^29,31,32^. In contrast, the QBSs in gapless BLG have not yet been fully studied. Although the QBSs under zero magnetic field have discussed in Ref.^26^, the evolution of QBSs under magnetic field has not yet been investigated.

Under the magnetic field, the electrons generally exhibits different phenomena on bilayer from monolayer graphene. For instance, the half-integer quantum Hall effect originated from the $\pi$ Berry phase has been discovered in graphene^3,21,33–35^, while a conventional integer quantum Hall effect associated with $2\pi$ Berry phase has been detected in BLG^19,33,36^. Though the evolution of QBSs in CNPJs of monolayer graphene under magnetic field has been studied^14,37^, it is unknown that how does the magnetic field affect the QBS spectra in bilayer graphene CNPJ, which may demonstrate different features compared with the case in monolayer graphene.

In this paper, we study the evolution of the QBSs spectra in BLG under the perpendicular magnetic fields using Wentzel–Kramers–Brillouin(WKB) approximation and the numerical method. We find that, at the presence of magnetic field, the QBSs of opposite angular momentum split and their splitting increases with the magnetic field strength. Furthermore, we demonstrate that weak magnetic fields can slightly shift the position of QBSs, while strong magnetic fields give rise to additional resonance peaks besides QBSs due to the Landau levels. These results provide comprehensive understanding of the evolution of quasi-bound states in CNPJ of BLG under the magnetic field.

This paper is organized as follows. In Section “Model and quasi-bound states spectrum”, we give an analytical solution for the quasi-bound states in a CNPJ of BLG under zero magnetic field, which are useful for the numerical and WKB analysis in the following sections. In Section “Energy spectra of quasi-bound states in magnetic fields”, we investigate the evolution of the QBSs spectra under the magnetic fields numerically and analyze the results using the semiclassical WKB approximation. In Section “Discussion”, we discuss the validity of our model, before summarizing our main results in Section “Summary”.

## Model and quasi-bound states spectrum

In this section, we consider the scattering of a plane wave electron on a CNPJ in BLG and calculate the local density of states (LDOS) of QBSs based on the two-band continuum Hamiltonian. Then we use the LDOS map to analyze how the QBSs’ properties change with different potential barrier radius and strength.

**Figure 1:**
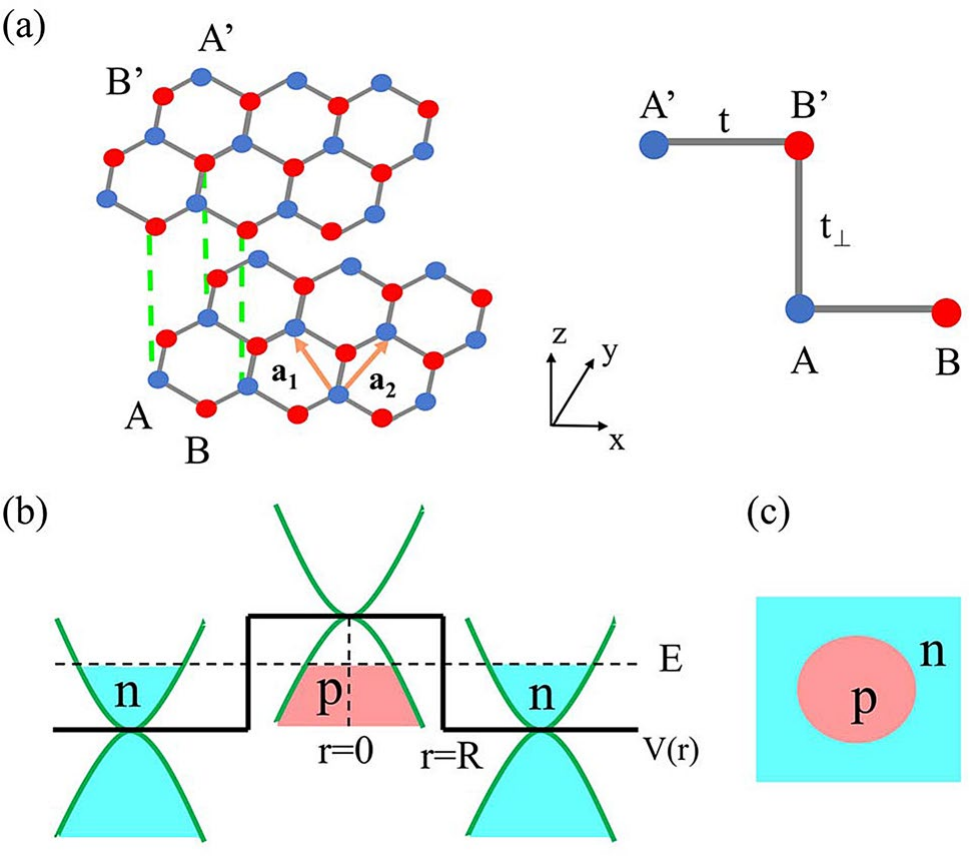
(**a**) Side view of the crystal of Bernal staking BLG, where $\mathbf{a} _{1}$, $\mathbf{a} _{2}$ are the basis vectors of monolayer graphene and *A*, *B*($A'$, $B'$) are two sublattices of each layer. (**b**) Illustration of Klein tunneling across a CNPJ of BLG with energy of *E* and potential of *V*(*r*). (**c**) Top view of the circular potential in BLG, where QBSs appear in the *p* region.

The Bernal ($A-B'$) stacked BLG is shown in Fig.  1a. Taking into account in-plane hopping parameter $\gamma {}_{AB}=\gamma _{A'B'}\equiv t$ and inter-layer coupling parameter $\gamma _{A'B}\equiv t_{\perp }$ for undoped BLG, four bands model can be obtained by considering one $2p_z$ orbital on each of the four atomic sites in the unit cell ($A, B, A', B'$)^2,23^. Near the Dirac point *K* and $K'$, the two low-energy bands are touched and can be approximated as $E_{\pm }\left( k\right) =\pm \frac{\hbar ^{2}k^{2}}{2m}$, where $m=\frac{\left| t_{\perp }\right| }{2v^{2}}$ is the effective mass, $v=10^{6}m/s$ is fermion velocity of electron, and $a\approx 1.42\mathring{A}$ is the nearest-neighbour distance.

We consider a CNPJ of gapless BLG and model a circular potential barrier with the step-like potential $V=V(r)\sigma _{0}=V_{0}\Theta (R-r)\sigma _{0}$, as shown in Fig.  1b,c. Focusing on the dynamics near a single Dirac point at K, the full two-band Hamiltonian is given by^38^:

1 $$\begin{aligned} H=H_{0}+V=\frac{1}{2m}\left( \begin{array}{cc} 2mV(r) &{} p_{-}^{2}\\ p_{+}^{2} &{} 2mV(r) \end{array}\right) , \end{aligned}$$

where $p_{\pm }=p_{x}\pm ip_{y}$. The validity of Eq. (1) are discussed in Section “Discussion”.

To solve this equation, we start by writing the canonical momentum operators as $p_{\pm }=\frac{\hbar }{i}e^{\pm i\phi }\left( \partial r\pm \frac{i}{r}\partial \phi \right)$. The Hamiltonian commutes with the pseudo angular momentum operator $J_{z}=L_{z}+\hbar \sigma _{z}$ due to the radially symmetric potential. Here, $L_{z}=({\mathbf {r}}\times {\mathbf {p}})_{z}$, and $\sigma _{z}$ is the third Pauli matrix. Then, we need look for eigenfunctions of $J_{z}=L_{z}+\hbar \sigma _{z}$ with eigenvalues $j=l+1=1,2,3$,…, where $l=0,1,2$,…. Assuming wavefunction is

2 $$\begin{aligned} \varPsi =e^{ij\phi }\left( \begin{array}{c} e^{-i\phi }\chi _{A}(r)\\ -e^{i\phi }\chi _{B}(r) \end{array}\right) =\left( \begin{array}{c} e^{i(j-1)\phi }\chi _{A}(r)\\ -e^{i(j+1)\phi }\chi _{B}(r) \end{array}\right) . \end{aligned}$$

where the phase factor $e^{\pm i\phi }$ is derived from the BLG Hamiltonian, the coupled eigen equations are obtained:

3 $$ \begin{aligned} \left( \partial ^{2}r+\frac{2j+1}{r}\partial r+\frac{j^{2}-1}{r^{2}}\right) \chi _{B}\left( r\right)  =\frac{2m\left( E-V\right) }{\hbar ^{2}}\chi _{A}\left( r\right) ; \end{aligned}$$

4 $$\begin{aligned} \left( \partial ^{2}r-\frac{2j-1}{r}\partial r+\frac{j^{2}-1}{r^{2}}\right) \chi _{A}\left( r\right)  =\frac{2m\left( E-V\right) }{\hbar ^{2}}\chi _{B}\left( r\right) . \end{aligned} $$

We solve Eqs. (3) and (4) by considering a scattering processes^25,39^, that an incident electron with energy *E* in BLG is scattered by a CNPJ created by a gate-induced circular potential barrier *V*(*r*). The incident plane wave in the *n* region can be written as a certain combination of cylindrical waves, then we utilize the scatter theory and the properties of and Bessel functions to get the wavefunctions outside the barrier (*n* region) as^25^:

5 $$\begin{aligned} \mathbf{h }_{j}^{(1)}\left( r,\phi \right) =\left[ \begin{array}{c} H_{j-1}^{(1)}\left( k_{n}r\right) e^{-i\phi }\\ \alpha 'H_{j+1}^{(1)}\left( k_{n}r\right) e^{i\phi } \end{array}\right] e^{ij\phi },\end{aligned}$$

6 $$\begin{aligned} \mathbf{h }_{j}^{(2)}\left( r,\phi \right) =\left[ \begin{array}{c} H_{j-1}^{(2)}\left( k_{n}r\right) e^{-i\phi }\\ \alpha 'H_{j+1}^{(2)}\left( k_{n}r\right) e^{i\phi } \end{array}\right] e^{ij\phi }, \end{aligned}$$

7 $$\begin{aligned} \mathbf{k }_{j}\left( r,\phi \right) =\left[ \begin{array}{c} K_{j-1}\left( k_{n}r\right) e^{-i\phi }\\ \alpha 'K_{j+1}\left( k_{n}r\right) e^{i\phi } \end{array}\right] e^{ij\phi }. \end{aligned}$$

Within the barrier (*p* region), the regular eigenfunctions of the Hamiltonian *H* with energy *E* are^25^

8 $$\begin{aligned} \mathbf{j }_{j}\left( r,\phi \right) =\left[ \begin{array}{c} J_{j-1}\left( k_{p}r\right) e^{-i\phi }\\ \alpha J_{j+1}\left( k_{p}r\right) e^{i\phi } \end{array}\right] e^{ij\phi }, \end{aligned}$$

9 $$\begin{aligned} \mathbf{i }_{j}\left( r,\phi \right) =\left[ \begin{array}{c} I_{j-1}\left( k_{p}r\right) e^{-i\phi }\\ \alpha I_{j+1}\left( k_{p}r\right) e^{i\phi } \end{array}\right] e^{ij\phi }. \end{aligned}$$

**Figure 2:**
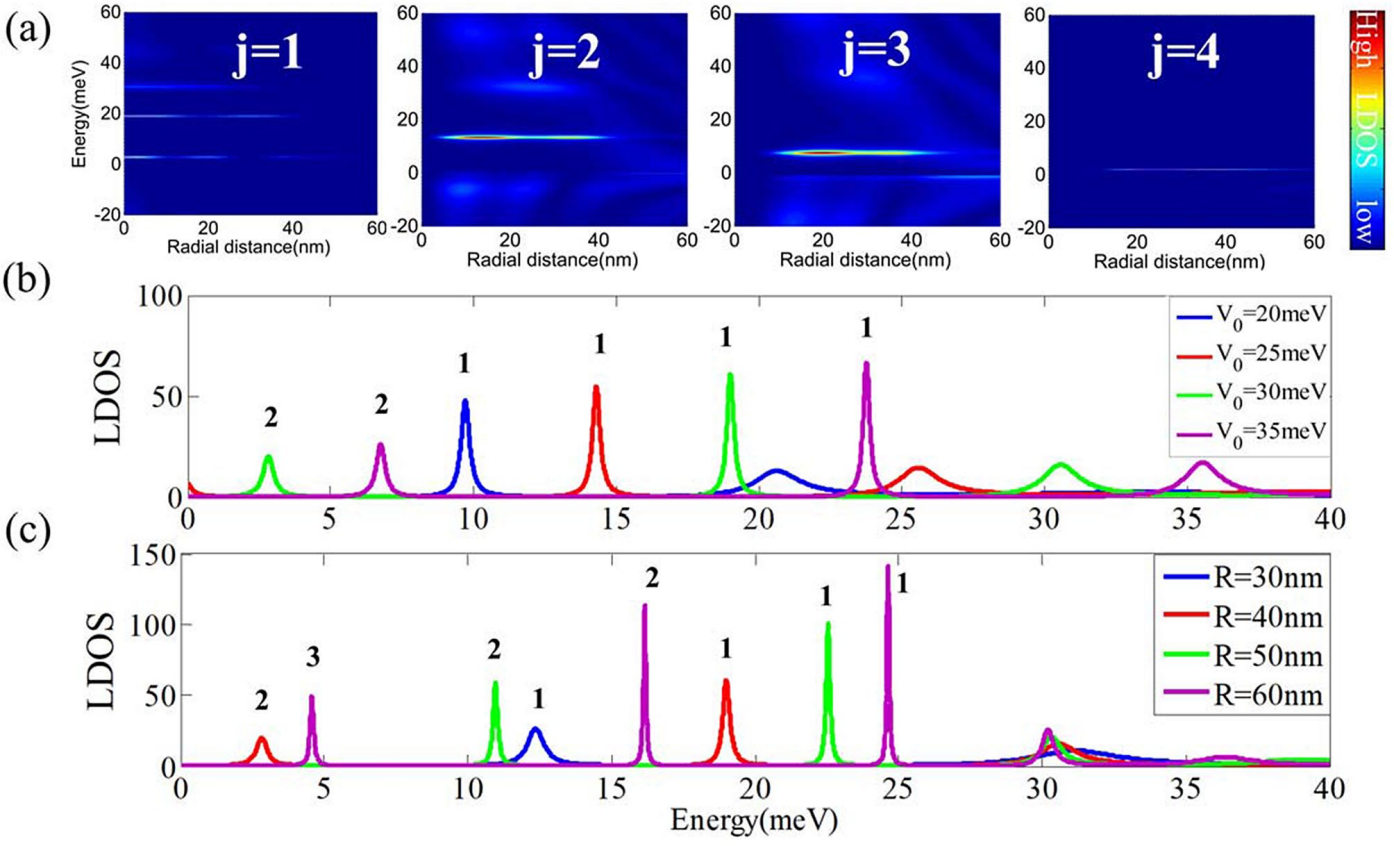
(**a**) LDOS maps for quasi-bound states in CNPJ of bilayer graphene with barrier radius $R=40$ nm, barrier potential $V_{0}=30$ meV for four angular momentum channels $j=1,2,3,4$. The QBSs energy levels decrease with *j*. (**b**) Peaks of LDOS represent the energy levels in $R=40$ nm BLG quantum dot for the $j=1$ mode at position $r_{0}=10$ nm, The different lines indicate different cases with barrier potentials $V_{0}=20$ meV (blue), 25 meV (red), 30 meV (green), 35 meV (purple), respectively. The energy levels of QBSs increases with the barrier potential $V_{0}$. (**c**) Peaks of LDOS represent the energy levels in $V_{0}=30$ meV BLG quantum dot for the $j=1$ mode at position $r_{0}=10$ nm, The different lines indicate different cases with barrier radius $R=30$ nm (blue), 40 nm (red), 50 nm (green), 60 nm (purple), respectively. The energy levels spacing of QBSs decreases with the barrier radius *R*. The numbers of peak in (**b**,**c**) represent the quantum number of energy levels.

These functions are simultaneously eigenfunctions of *H* and $J_{z}$, with eigenvalues *E* and $\hbar j$, respectively. Here, $H_{j}^{(1)}$, $H_{j}^{(2)}$ are the Hankel functions of first and second kind, $K_{j}$, $I_{j}$ are the modified Bessel functions, $k_{n}=\sqrt{2mE}/\hbar$, $k_{p}=\sqrt{2m(E-V_{0})}/\hbar$ denote wavevectors in the *n*, *p* region, respectively. And we use $\alpha '=sgn(E)$ and $\alpha =sgn(E-V_{0})$ to ensure the proper signs for electrons and holes.

In the *n* region, $H_{j}^{(1)}$, $H_{j}^{(2)}$, and $K_{j}$ are effective eigenfunctions which are bounded for large arguments, and we disregard other eigenfunctions that diverge for large arguments. Similarly, in the *p* region, we consider $J_{j}$ and $I_{j}$ but ignore the other eigenfunctions which are divergent at the origin. Thus, the complete wavefunction can be written as^25^

10 $$\begin{aligned} \psi _{j}^{(n)}= & {} \mathbf{h }_{j}^{(2)}+S_{j}\mathbf{h }_{j}^{(1)}+A_{j}\mathbf{k }_{j}, \end{aligned}$$

11 $$\begin{aligned} \psi _{j}^{(p)}= & {} B_{j}\mathbf{j }_{j}+C_{j}\mathbf{i }_{j}, \end{aligned}$$

in the *n* and *p* region, respectively. The coefficients $S_{j}$, $A_{j}$ , $B_{j}$ and $C_{j}$ can be obtained from the boundary conditions at the interface of the CNPJ: the wavefunctions and their derivatives at $r=R$ are continuous

12 $$\begin{aligned} \psi _{j}^{(n)}|_{r=R}= & {} \psi _{j}^{(p)}|_{r=R}, \end{aligned}$$

13 $$\begin{aligned} \frac{\partial \psi _{j}^{(n)}}{\partial r}|_{r=R}= & {} \frac{\partial \psi _{j}^{(p)}}{\partial r}|_{r=R}. \end{aligned}$$

Therefore, we can calculate the local density of states by $LDOS(j,r,E) \propto \left| \varPsi \left( r,E\right) \right| ^{2}$. In the *n* region,

14 $$\begin{aligned} \left| \psi ^{^{\left( n\right) }}\left( j,m^{*},r,E\right) \right| ^{2}= \left( D_{-}\left( k_{n}r\right) \right) ^{2}+\left( D_{+}\left( k_{n}r\right) \right) ^{2}. \end{aligned}$$

where

15 $$\begin{aligned}D_{-}\left( k_{n}r\right) =H_{j-1}^{(1)}\left( k_{n}r\right)  +S_{j}H_{j-1}^{(2)}\left( k_{n}r\right) +A_{j}K_{j-1}\left( k_{n}r\right) , \end{aligned} $$

16 $$\begin{aligned} D_{+}\left( k_{n}r\right) =H_{j+1}^{(1)}\left( k_{n}r\right)  + S_{j}H_{j+1}^{(2)}\left( k_{n}r\right) +A_{j}K_{j+1}\left( k_{n}r\right) , \end{aligned} $$

In the *p* region,

17 $$\begin{aligned} \left| \psi ^{\left( p\right) }\left( j,m,r,E\right) \right| ^{2}=\left( F_{-}\left( k_{p}r\right) \right) ^{2}+\left( F_{+}\left( k_{p}r\right) \right) ^{2}. \end{aligned}$$

where

18 $$\begin{aligned} F_{-}\left( k_{p}r\right)= & {} B_{j}J_{j-1}\left( k_{p}r\right) +C_{j}I_{j-1}\left( k_{p}r\right) , \end{aligned}$$

19 $$\begin{aligned} F_{+}\left( k_{p}r\right)= & {} B_{j}J_{j+1}\left( k_{p}r\right) +C_{j}I_{j+1}\left( k_{p}r\right) \end{aligned}$$

Figure 2a depicts the *LDOS*(*j*, *r*, *E*) for resonant states at different angular momenta. As *j* increases, the resonant modes shift from the center of the quantum dot and gradually move outward, which acts similarly to those in monolayer graphene^10^. However, the QBSs in BLG for $l=0$ are narrower compared to the $l=1$, $l=2$ modes. This feature is different from monolayer graphene due to their different band structures^10,26^. The QBSs spectra can be measured experimentally via STM. Besides, we notice that the QBSs are formed in the *p* region, which can be regarded as BLG quantum dots.

Figure 2b and c show the QBSs energy levels change with different potential barrier radius *R* and strength $V_{0}$ for the $j=1$ mode at position $r_{0}=10$ nm. Here, the peaks of LDOS in the *p* region represent the energy levels of QBSs. In general, the higher barrier potential trap more QBSs along with the wider energy spacings. Likewise, larger bilayer graphene quantum dot can trap more QBSs with the narrower energy spacing. Additionally, the trapping time can be obtained through half-width of energy levels by $\tau =\frac{\hbar }{\triangle E}$. For larger *R* and higher $V_{0}$, QBSs can be trapped longer. These results suggest that we can confine specific energies and angular momentum modes by adjusting the potential size and depth. Note that the above calculations neglect valley mixing, for reasons explained in Section “Discussion”.

## Energy spectra of quasi-bound states in magnetic fields

In this section, we numerically solve the radial equation for a CNPJ of BLG in the presence of an external perpendicular magnetic field. Following, we focus on the case that the magnetic field is not sufficiently strong to make system fully evolve into Landau levels. In order to provide a simpler and more intuitive physical picture, we also give a semiclassical analysis of QBSs based on the WKB approximation at the end of this section.

When a magnetic field is applied perpendicularly on the graphene surface, the orbital motion of electrons in two-dimension is quantized and the spectrum becomes discrete, called Landau levels. These Landau levels inevitably have an influence on the QBSs. Under the magnetic field, quasi-particles in the low-energy regime can be characterized by the Hamiltonian in Eq. (1) via making the substitution $\mathbf {p}\rightarrow \mathbf {p}+e\mathbf {A}$, where the radial gauge $\mathbf {A}=\left( -\frac{By}{2},\frac{Bx}{2},0\right)$. Writing the canonical momentum operators as

20 $$\begin{aligned} p^{+}= & {} ie^{i\phi }\left( i\hbar \partial r-i\frac{\hbar }{r}\partial \phi +\frac{eBr}{2}\right) \end{aligned}$$

21 $$\begin{aligned} p^{-}= & {} -ie^{i\phi }\left( i\hbar \partial r-i\frac{\hbar }{r}\partial \phi +\frac{eBr}{2}\right) , \end{aligned}$$

the corresponding radial equations become

22 $$\begin{aligned}  \partial ^{2}r\chi _{B}(r)+\left( \frac{2j+1}{r}+\frac{eBr}{\hbar }\right) \partial r\chi _{B}(r) +\left( \frac{j^{2}-1}{r^{2}}+\frac{e^{2}B^{2}r^{2}}{4\hbar ^{2}}+\frac{eB(j+1)}{\hbar }\right) \chi _{B}(r) =\frac{2m\left( E-V\right) }{\hbar ^{2}}\chi _{A}(r) \end{aligned} $$

23 $$\begin{aligned} \partial ^{2}r\chi _{A}(r)-\left( \frac{2j-1}{r}+\frac{eBr}{\hbar }\right) \partial r\chi _{A}(r)  +\left( \frac{j^{2}-1}{r^{2}}+\frac{e^{2}B^{2}r^{2}}{4\hbar ^{2}}+\frac{eB(j-1)}{\hbar }\right) \chi _{A}(r)  =\frac{2m\left( E-V\right) }{\hbar ^{2}}\chi _{B}(r). \end{aligned}$$

Next, according to these coupling equations, we solve the quasi bound states under the magnetic field with numerical method and give a semiclassical analysis based on the WKB approximation.

### Numerical solution under magnetic fields

To obtain the QBSs under the magnetic field, we start by solving the Eqs. (22) and (23) via the two sides finite difference method discretized in 600 sites in the interval $0^{+}<r<L$ ($L>R$ is truncation position). The initial wavefunction of finite difference method is $\psi _{0}$ at $r\simeq 0^{+}$, while $\psi _{L}$ at $r=L$. According to the finite difference method, $\psi _{0}$ and $\psi _{L}$ evolve with the formula from two sides to n–p junction boundary $r=R$. Then, analogy to the analytic method at $B=0$ in Section “Model and quasi-bound states spectrum”, we apply the boundary condition of $\psi _{0}$ and $\psi _{L}$ at $r=R$ to obtain the new coefficients under the magnetic field^11,26,35^. To be specific, at $r\simeq 0^{+}$ side, owing to $\frac{eBr}{\hbar }\ll \frac{2j-1}{r}$ and $\frac{e^{2}B^{2}r^{2}}{4\hbar ^{2}}+\frac{eB\left( j-1\right) }{\hbar }\ll \frac{j^{2}-1}{r^{2}}$, Eqs. (22) and (23) can be reduced to Eqs. (3) and (4) by neglecting the magnetic terms. Consequently, we can directly use the analytical solution of zero magnetic field case, a set of Bessel function [Eqs. (5–9)], as $\psi _{0}$. For other side $r=L$, the magnetic field become dominant to give rise to the Landau level spectrum. Thus, we can not directly utilize the Eqs. (5–9) as initial wavefunctions at $r=L$. Here, we consider the low magnetic field case, and under the influence of disorder the wave function acquires a Lorentzian weight $\sum _{n}\frac{\triangle ^{2}}{\left( \varepsilon -\varepsilon _{n}\left( B\right) \right) ^{2}+\triangle ^{2}}$ on the zero magnetic field $\psi _{0}$ to produce $\psi _{L}$. This treatment continuously returns to the zero magnetic field case, and reflects the Landau levels effect on large distance induced by magnetic field. Note that this approximation works well when the magnetic length $l_{B}=\sqrt{\frac{\hbar }{eB}}$ is comparable to barrier radii *R*. Because small $l_{B}$ will make the whole system evolves into Landau levels, which beyond our research. Here, $\triangle =0.5$ meV is a broadening parameter from disorder scattering, $\varepsilon _{n}\left( B\right) =\hbar \omega _{c}\sqrt{n(n-1)}$ is spectrum of BLG under magnetic field^2^, and $\omega _{c}=\frac{eB}{mc}$ is the cyclotron frequency of non-relativistic electrons with effective mass *m*. Moreover, the results are proved to be insensitive to the details of the cutoff, as an example, we take a cutoff at $L=1.5R$ below.

**Figure 3:**
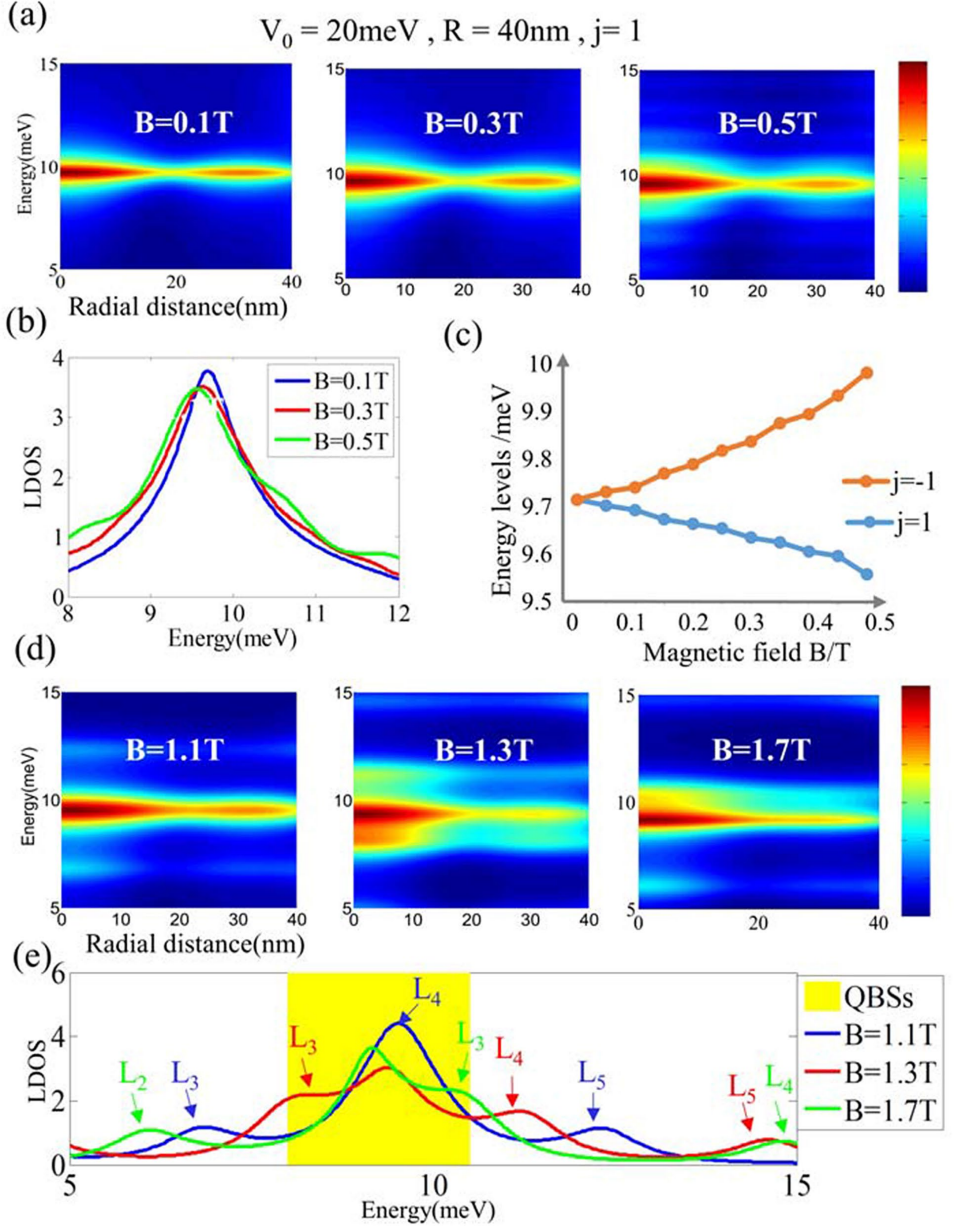
(**a**,**d**) show the evolution of the LDOS under relatively weak and strong magnetic fields, respectively. (**b**,**e**) show the detail effect on energy levels, respectively. Here, we plot the LDOS in logarithmical scale to show the magnetic field effect clearer, and the peaks in the yellow region indicate the QBSs energy levels. (**c**) Show the QBS energy levels for $j=1$ and $j=-1$ as the function of magnetic field strength *B*. And the magnetic field lift the energy levels for $j=-1$ mode and pull down those of the $j=1$ mode. (**e**), the arrows labeled $L_{x}$ mark the Landau levels. Taking $B=1.7$ T (green) as an example, $L_{2}$, $L_{3}$ and $L_{4}$ are BLG Landau levels with quantum numbers $n=2, 3, 4$ and the QBSs energy level is close to $L_{3}$.

At relatively weak magnetic field $B<0.5$ T, magnetic field only slightly shifts QBSs, as shown in Fig. 3a,b. Here, we plot the LDOS in logarithmic scale to clearly display the subpeaks induced by weak magnetic field. The energy shift is about 0.06 meV for 0.1 T, which evaluated from Fig. 3c. Furthermore, we notice the system has time-reversal symmetry at $B=0$ T, which guarantees the degeneracy pair $E_{K}(j)=E_{K}(-j)$. However, the finite magnetic field break time-reversal symmetry of the system. The degenerate states of opposite angular momentum separate and the energy splitting enlarges as *B* increases. Specifically, with *B* increasing, the energies of QBSs for $j=+n$ mode decrease while for $j=-n$ increase.

For relatively strong magnetic field, we plot the evolution of QBSs spectra under $B=1.1$ T, 1.3 T and 1.7 T, as shown in Fig. 3d,e. Comparing with the weak magnetic case, a stronger magnetic field have a more obvious effect on the QBSs due to the appearance of Landau levels and this effect becomes larger as *B* increases. The Landau levels appear next to the QBSs energy levels, and it seems to be no arresting interplay between them from numerical results. The QBSs and Landau levels do not merge or simply repel but coexist in this transition region. The LDOS peaks are the superposition between the confined state and the Landau levels. If we continue to increase the magnetic field *B*, when it exceeds the critical magnetic field $B_{c}$, the QBSs will disappear and the whole system will evolves into Landau levels. Here, $B_{c}$ can be evaluated from the magnitude of Landau levels and QBSs energy, and it is about 3 T at $R=40$ nm for $j=1$ mode. Therefore, the QBSs in a CNPJ of BLG can be tuned by adjusting the magnetic field strength as well.

### WKB approximation for zero and weak magnetic fields

**Figure 4:**
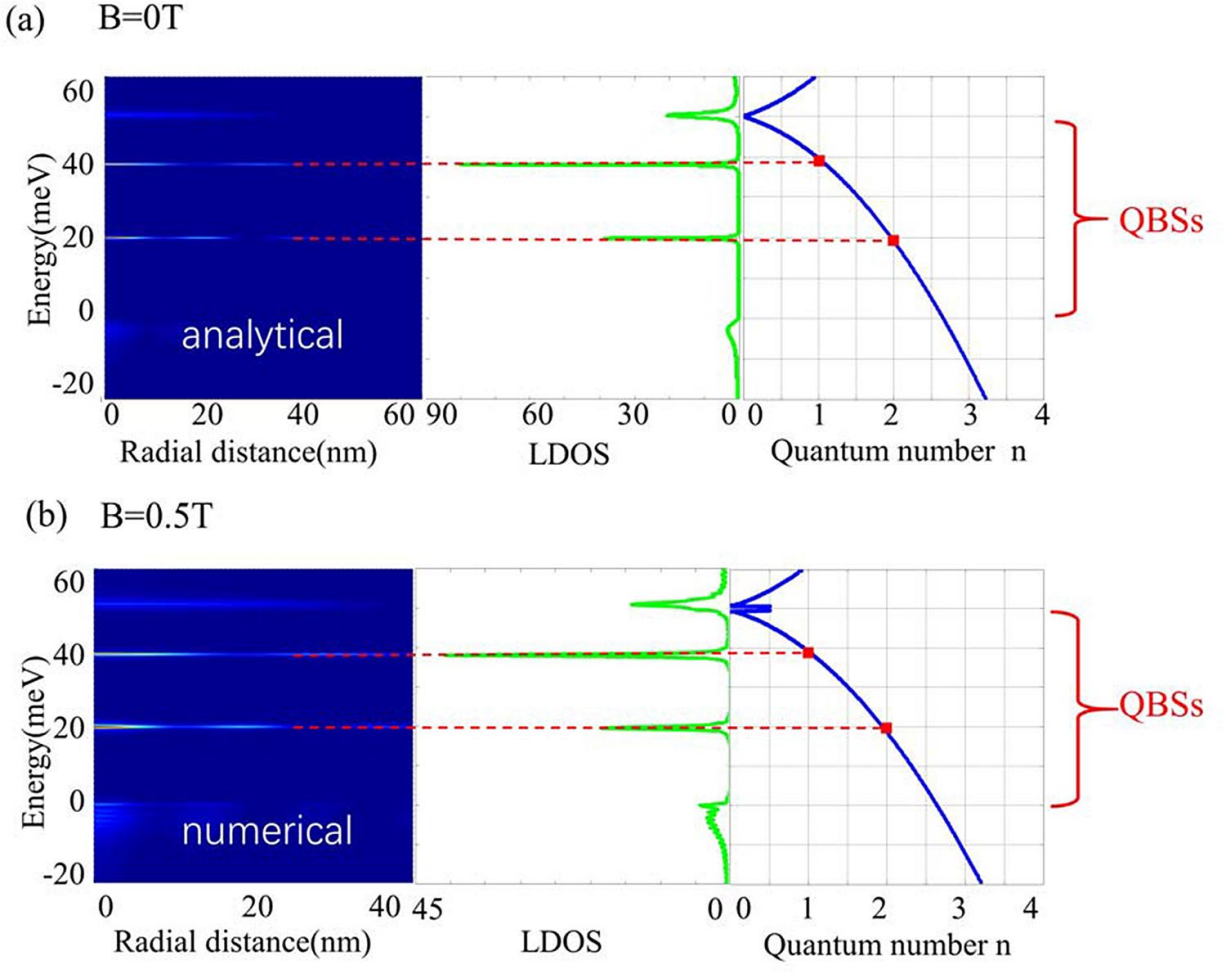
WKB analysis for quasi-bound states in CNPJ of BLG (**a**) without and (**b**) with magnetic field. The blue curve is from quantization condition Eq. (38), the red dots on blue curve corresponds to the peaks of LDOS, justifying the consistency between WKB solution and analytical (numerical) results.

Aforementioned numerical results cannot directly show the impact of particular parameters on the QBSs. To obtain a better understanding of numerical results, we can analyze the QBSs with semiclassical method based on WKB approximation. Reconsidering Eqs. (22) and (23), we firstly rewrite these equations in the matrix form^37,40,41^:

24 $$\begin{aligned} \chi ''+\frac{1}{\hbar }F\chi '+\frac{1}{\hbar ^{2}}G\chi =0, \end{aligned}$$

where

25 $$\begin{aligned} \chi= & {} \left( \begin{array}{c} \chi _{A}\\ \chi _{B} \end{array}\right) , \end{aligned}$$

26 $$\begin{aligned} F\left( j,B,r\right)= & {} \left( \begin{array}{cc} -\hbar \left( \frac{2j-1}{r}+\frac{eBr}{\hbar }\right) &{} 0\\ 0 &{} \hbar \left( \frac{2j+1}{r}+\frac{eBr}{\hbar }\right) \end{array}\right) , \end{aligned}$$

27 $$\begin{aligned} G\left( j,B,r\right)= & {} \left( \begin{array}{cc} \hbar ^{2}G_{+}\left( j,B,r\right) &{} -2m\left( E-V_{0}\right) \\ -2m\left( E-V_{0}\right) &{} \hbar ^{2}G_{-}\left( j,B,r\right) \end{array}\right) , \end{aligned}$$

28 $$\begin{aligned} G_{\pm }\left( j,B,r\right)= & {} \frac{j^{2}-1}{r^{2}}+\frac{e^{2}B^{2}r^{2}}{4\hbar ^{2}}+\frac{eB\left( j\pm 1\right) }{\hbar }. \end{aligned}$$

Then, we suppose

29 $$\begin{aligned} \chi (r)=\psi (r)exp\left\{ i\int y\left( r\right) dr\right\} . \end{aligned}$$

where *y*(*r*) can be written in the form of Taylor expansion

30 $$\begin{aligned} y(r)=\sum _{n=0}^{\infty }\hbar ^{n}y_{n}(r), \end{aligned}$$

So, we obtain the determinant of the first-order quasi-classical momentum $y_{0}=q_{B}$,

31 $$\begin{aligned} \left| \begin{array}{cccc} L_{-}(j,B,r) &{} -2m\left( E-V_{0}\right) \\ -2m\left( E-V_{0}\right) &{} L_{+}(j,B,r) \end{array}\right| =0, \end{aligned}$$

where

32 $$\begin{aligned} L_{\pm }(j,B,r)=-q_{B}^{2}\pm iq_{B}\left( \frac{2j\pm 1}{r}+\frac{eBr}{\hbar }\right) +G_{\pm }\left( j,B,r\right) . \end{aligned}$$

Here, in order to get a nice formula of $q_{B}$, we simplify the calculation by replacing $L_{\pm }(j,B,r)$ with $L^{'}_{\pm }(j,B,r)$:

33 $$\begin{aligned} \begin{aligned} L^{'}_{\pm }(j,B,r)&=-q_{B}^{2}\pm iq_{B}\left( \frac{2j}{r}+\frac{eBr}{\hbar }\right) +\frac{j^{2}-1}{r^{2}}\\&+\frac{e^{2}B^{2}r^{2}}{4\hbar ^{2}}+\frac{eBj}{\hbar }. \end{aligned} \end{aligned}$$

Then, we have the momentum $q_{B}$:

34 $$\begin{aligned} q_{B}^{2}(r)=-\frac{eB}{\hbar }j-\frac{1+j^{2}}{r^{2}}-\frac{e^{2}B^{2}r^{2}}{4\hbar ^{2}}+\frac{M\left( j,B,r\right) }{r^{2}}, \end{aligned}$$

where

35 $$\begin{aligned} M\left( j,B,r\right)  = \sqrt{4j^{2}+\frac{4eBjr^{2}}{\hbar }+\frac{e^{2}B^{2}r^{4}}{\hbar ^{2}}+4m^{2}\left( E-V_{0}\right) ^{2}r^{4}}. \end{aligned} $$

Corresponding to the solution of quasi-bound states, $q_{B}^{2}(r)>0$ give classically allowed region $r_{B}<r<R$^42,43^, where

36 $$\begin{aligned} r_{B}=\frac{\sqrt{-\frac{2eBj}{\hbar }+4m\left( E-V_{0}\right) -N(j,B)}}{\frac{eB}{\hbar }}, \end{aligned}$$

with

37 $$\begin{aligned} N\left( j,B\right)  =\sqrt{\frac{4e^{2}B^{2}\left( 1-j^{2}\right) }{\hbar ^{2}}+\left[ \frac{2eBj}{\hbar }+4m\left( E-V_{0}\right) \right] ^{2}}, \end{aligned} $$

Then applying Bohr–Sommerfeld quantization condition

38 $$\begin{aligned} \int _{r_{B}}^{R}q_{B}(r)dr=n\pi +\delta , \end{aligned}$$

we can obtain the relation between energy level $E_{n,j}$ of QBSs and quantum number *n*. Here, we take phase factor $\delta = 0$, because the step-like circular potential can be regarded as two vertical barrier potential in radial direction, and this geometrical shape of boundary corresponds to $\delta = 0$^44^. In Fig. 4, the blue curves in rightmost panels depict the relation of $E_{j}$ and *n*, and the red dots represent the position of QBSs. Setting $B=0$ T in the above formula, the energy levels of QBSs are consistent with the rigorous results shown in Section “Model and quasi-bound states spectrum”. Likewise, at relatively weak magnetic field $B<0.5$ T, the WKB solution are in accordance with the numerical results in Section “Numerical solution under magnetic fields”. These results verify the availability of the WKB approximation. Thus, WKB approximation provides an easier way for predicting the QBSs energies.

**Figure 5:**
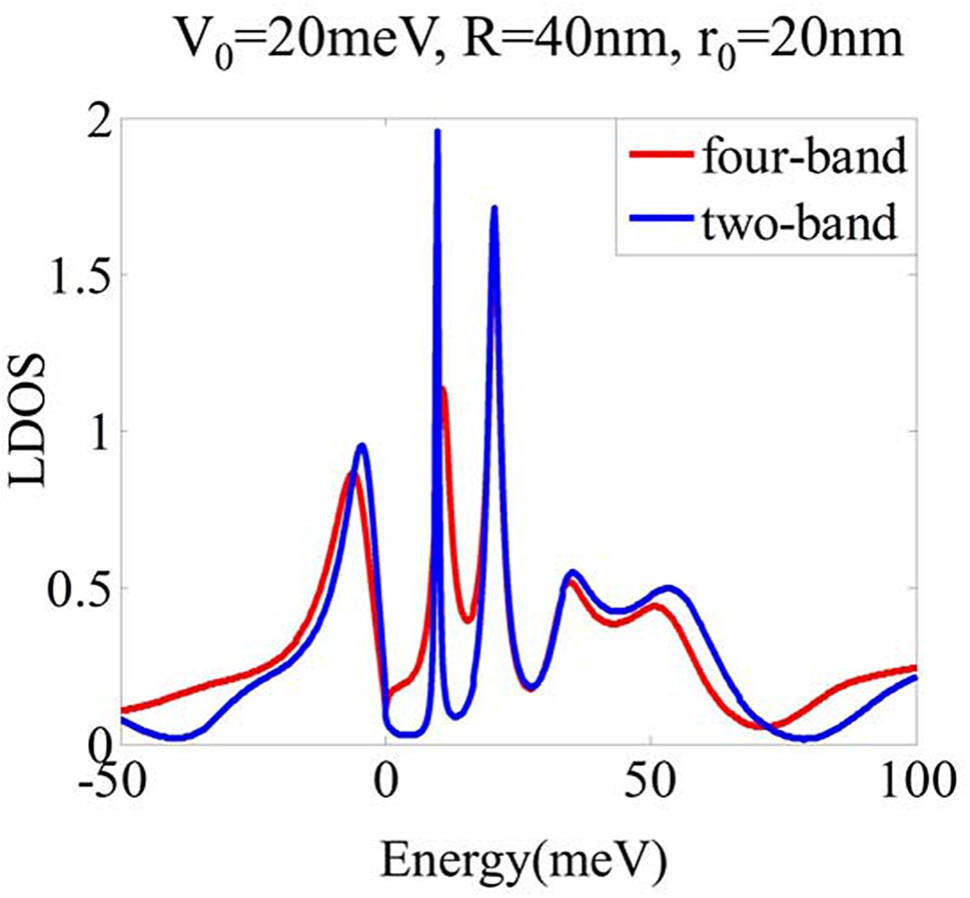
Comparison of the LDOS as functions of energy derived from the two-band and four-band model.

## Discussion

Throughout the above analysis of CNPJ, we have modeled the electrostatic potential as a step-like function of position. This assumption is justified if $a\ll R$ ensures the absence of inter-valley scattering at the interface, where *a* is the lattice constant and *R* is the characteristic length representing the width of the transition region between the junction’s *n* and *p* sides. The inter-valley scattering is inevitable in experiments, but as investigated in Fig. 3 of Ref.^15^, the authors demonstrated that the inter-valley scattering caused by the step potential is very weak and QBSs are nearly insensitive to the smoothness of boundary. These results are in good agreement with those of experiments^10,13,14^. These previous investigations justify the validity of our approximation.

Regarding the feasibility of the two-band Hamiltonian in Eq. (1), there are two points needs to address. Firstly, we require $E_{F}<t_{\perp }/2\approx 200$ meV to ensure the quadratic dispersion relation holds. Secondly, we neglect the trigonal warping term. Thus, our model is reliable for quasi-particles in the energy regime under 200 meV^25^. Furthermore, we calculate the LDOS as a function of energy derived from the two-band Hamiltonian and four-band Hamiltonian to verify the validity of the two-band Hamiltonian in Eq. (1), as shown in Fig. 5.

Besides, the QBSs of CNPJ in our study differ from those of Coulomb potential. The QBSs of long range Coulomb potential exhibit the dramatic property of discrete scale invariance^45,46^. In contrast, the circular potential added on our system is confined potential and the QBSs of them don’t show the discrete scale invariance. Thus, the results of these two kinds of potential are different in the qualitative and quantitative studies. Moreover, the construction of circular np junction in graphene has already been achieved experimentally, and our theoretical results can be useful for qualitative analysis to them.

## Summary

In this paper, we have studied the quasi-bound states in a circular n–p junction of bilayer graphene and their evolution under the magnetic field numerically. We have shown that the quasi-bound states spectra can be controlled by adjusting the potential barrier radius and strength. These energy spectra are quantitatively different from those for monolayer graphene due to different band structures. We also have demonstrated that the energy level degeneracy of opposite angular momentum states breaks under magnetic field and the energy splitting enlarges as magnetic field strength increases. Moreover, applying weak magnetic fields on system leads to slight shift of quasi-bound states. While the strong magnetic fields induce additional resonances beside the quasi-bound states. These additional resonances originate from the Landau levels. The evolution of quasi-bound state spectra under magnetic field is also supplemented with semiclassical analysis based on the WKB approximation. Our results are highly relevant to recent experiments and can be verified in STM measurement.
